# Progesterone Signaling in Endometrial Epithelial Organoids

**DOI:** 10.3390/cells11111760

**Published:** 2022-05-27

**Authors:** Sylvia C. Hewitt, San-pin Wu, Tianyuan Wang, Steven L. Young, Thomas E. Spencer, Francesco J. DeMayo

**Affiliations:** 1 Pregnancy and Female Reproduction, Reproductive and Developmental Biology Laboratory, National Institute of Environmental Health Sciences (NIEHS), Research Triangle Park, NC 27709, USA; sylvia.hewitt@nih.gov (S.C.H.); steve.wu@nih.gov (S.-p.W.); 2 Integrative Bioinformatics Support Group, National Institute of Environmental Health Sciences (NIEHS), Research Triangle Park, NC 27709, USA; tianyuan.wang@nih.gov; 3 Department of Obstetrics and Gynecology, University of North Carolina at Chapel Hill, Chapel Hill, NC 27599, USA; steven_young@med.unc.edu; 4 Department of Obstetrics, Gynecology and Women’s Health, University of Missouri, Columbia, MI 65211, USA; spencerte@missouri.edu

**Keywords:** progesterone, estrogen, endometrium

## Abstract

For pregnancy to be established, uterine cells respond to the ovarian hormones, estrogen, and progesterone, via their nuclear receptors, the estrogen receptor (ESR1) and progesterone receptor (PGR). ESR1 and PGR regulate genes by binding chromatin at genes and at distal enhancer regions, which interact via dynamic 3-dimensional chromatin structures. Endometrial epithelial cells are the initial site of embryo attachment and invasion, and thus understanding the processes that yield their receptive state is important. Here, we cultured and treated organoids derived from human epithelial cells, isolated from endometrial biopsies, with estrogen and progesterone and evaluated their transcriptional profiles, their PGR cistrome, and their chromatin conformation. Progesterone attenuated estrogen-dependent gene responses but otherwise minimally impacted the organoid transcriptome. PGR ChIPseq peaks were co-localized with previously described organoid ESR1 peaks, and most PGR and ESR1 peaks were in B (inactive) compartment regions of chromatin. Significantly more ESR1 peaks were assigned to estrogen-regulated genes by considering chromatin loops identified using HiC than were identified using ESR1 peak location relative to closest genes. Overall, the organoids model allowed a definition of the chromatin regulatory components governing hormone responsiveness.

## 1. Introduction

The female reproductive system encompasses anatomical structures, physiological and cellular processes, and external environmental signals working in a coordinated manner. In order to prepare the uterus for pregnancy, estrogen and progesterone are released from the ovary to act on the uterine endometrium, which consists of epithelial and stromal cells lining the uterus. A receptive endometrium will allow attachment and implantation of embryos [1]. The endometrium is comprised of multiple kinds of cells, including two specialized types of epithelial cells: luminal epithelial cells that line the uterine lumen and are the site of initial attachment of the embryo, and glandular epithelial cells that form tubes originating at the lumen and secrete cytokines and other substances needed for pregnancy [2]. Stromal fibroblast cells that are adjacent to the lumen and surround the glands undergo progesterone-dependent decidualization to support early pregnancy. Integrated signaling between stroma and epithelial cells establishes the “window of implantation” in optimally receptive endometrium. Estrogen and progesterone affect both endometrial cell types, with estrogen stimulating cell growth and with progesterone attenuating growth and establishing a secretory milieu. If pregnancy is not established, hormone levels decrease, and the endometrium is shed by menstruation [3].

Estrogen and progesterone impact intracellular processes via nuclear hormone receptor family members, the estrogen receptor (ESR) and progesterone receptor (PGR) [4,5]. Nuclear receptor proteins share multiple structural domains that encode activities intrinsic to their mechanism of action of binding to hormones and regulating transcription rates of genes. The central functions of the receptors are high affinity and specificity interaction with hormones via their ligand binding domain, interaction with specific DNA motifs via their DNA-binding domain, and interaction with chromatin-modifying transcriptional co-regulatory factors via activation functions (AFs) in their N-terminal A/B domain as well as within the ligand binding domain [5,6]. Two isoforms of PGR, PGR-A, and PGR-B, are produced from the PGR gene from two different promoters; both PGR isoforms are present in reproductive tissues [6]. PGR is constitutively expressed in endometrial epithelial cells and increases in uterine stromal cells as estrogen rises just prior to ovulation so that when progesterone increases after ovulation occurs, the endometrium can rapidly sense and respond to fluctuations in ovarian hormones by altering transcriptional programs to produce optimal conditions for pregnancy [6]. Progesterone regulates the level of estrogen activity within endometrial epithelial cells and, in particular, inhibits estrogen-stimulated epithelial cell growth, which is essential for implantation to occur [7]. Endometrial epithelial cell PGR expression decreases while FOXO1 trans-locates into the nucleus, leading to growth arrest [8]. Mice lacking PGR in the whole uterus or only in the uterine epithelial cells lack the ability to implant embryos and exhibit inappropriate epithelial proliferation [9,10,11]. These observations emphasize the key role of epithelial PGR in the establishment of receptivity. Conversely, mouse models with persistent epithelial PGR-A or PGR-B overexpression are unable to establish a receptive uterus [12,13], which illustrates the importance of appropriate dynamics of epithelial cell PGR levels for fertility. In women, progesterone is important not just for fertility but also for general endometrial health. Decreased progesterone signaling contributes to endometrial hyperplasia and cancer [14], and progesterone resistance in eutopic endometrium has been implicated in endometriosis [15].

PGR alters gene transcription by interacting with hormone-responsive enhancer (HRE) motifs in regions where pioneer factors, such as members of the Forkhead or GATA transcription factor families, have increased the accessibility of the chromatin [16]. When PGR binds progesterone, the structure within its ligand binding domain shifts, allowing interaction with histone-modifying transcriptional co-regulators, thereby increasing the accessibility of the chromatin and enhancing rates of transcription. Using ChIPseq, the sites of receptor/chromatin interaction, or cistrome, can be determined in particular cells and tissues. The PGR cistrome of mouse uterine tissue [13,17] and human endometrial biopsies [18] have been described and indicate that PGR binds near genes, as well as in distal enhancers. These distal sites are called enhancers because they interact with numerous transcription factors and histones with activating modifications to enhance gene transcription. Although enhancers can be located hundreds of kilobases (kb) from genes, enhancers and genes are brought into proximity within the nucleus by the 3-dimensional structure of chromatin. For example, in the mouse uterus, a distal PGR-binding enhancer 19 kb 5′ of the Indian hedgehog (*Ihh*) gene promoter was shown to be essential both for basal uterine expression of *Ihh* and its regulation by progesterone [19]. HiC is a technique that allows evaluation of 3D chromatin structure within cells and has revealed that at >100 kb resolution, chromatin is within A (inactive) and B (inactive) compartments; within compartments, at >10 kb resolution, distal chromatin locations interact via topologically associated domains (TADs) and loops [20]. These TAD and loop structures bring distal enhancers into proximity with genes. HiC of mouse uterine chromatin [21] and of endometrial cancer cells [22] has been described. Chromatin conformation of normal human uterine epithelial cells has not been reported but would be valuable for finding ESR1 and PGR binding enhancer interactions with the target gene controlling epithelial receptivity. In order to investigate the structure of endometrial epithelial chromatin we conducted a genome wide chromatin conformation capture, HiC on human organoids.

Many of the molecular details of progesterone signaling have been derived from mouse models, which allow both genetic manipulations to assess the roles of progesterone response components as well as treatments with exogenous substances. The development of models to similarly interrogate and define mechanisms in human uterine tissue is more challenging due to the invasive method required to obtain endometrial samples. Studies in cultured stromal cells are one key model, as they are amenable to mechanistic studies; however, in vitro models in which to study epithelial cells have proven challenging. Recently, however, innovative organoid culture systems have been developed that provide a platform to study processes intrinsic to endometrial epithelial cells [23,24,25].

Here, we assessed the progesterone-induced responses of human endometrial epithelial organoids by examining their transcriptome and PGR cistrome. We integrated these findings, and our previously published estrogen-induced transcriptional responses and ESR1 cistrome of human endometrial epithelial organoids [26], with HiC analysis of the 3D arrangement of human endometrial epithelial organoid chromatin to find candidate ESR1 or PGR gene interactions.

## 2. Materials and Methods

### 2.1. Organoid Culture

Organoid culture, isolation, and analysis of RNA and chromatin were described previously [26]. Organoids were plated and allowed to form for 4 days. They were then divided into 4 treatment groups as follows: Vehicle: 5 additional days treatment with no hormones, E2: 5 additional days of treatment with 10 nM estradiol (E2), E2+MPA: 2 additional days of treatment with 10 nM E2 followed by 3 days of treatment with 10 nM E2 and 1 µM medroxyprogesterone acetate (MPA), RU486+E2+MPA: 2 additional days of treatment with 10 nM E2 followed by 3 days of treatment with 10 nM E2, 1 µM MPA and 1 µm RU486. On the final day, 6 h after change to fresh media with hormones, organoids were collected for RNA isolation. For chromatin and protein isolation, only the E2+MPA treatment was used, and organoids were collected 1 h after the media change on the final day.

### 2.2. RNA and Chromatin Analyses

RNA was isolated using Trizol (Life Technologies Inc., Carlsbad, CA, USA) and analyzed by RT-PCR or RNAseq as previously described [26]. For RT-PCR, cDNA was prepared using Superscript II (Invitrogen, Carlsbad, CA, USA) with Random Hexamers (Invitrogen, Waltham, MA, USA), as previously described [27,28]. Sequences of primers (Sigma, St. Louis, MO, USA) used are: IHH F-GACCGCGACCGCAATAAGTA R-TGGGCCTTTGACTCGTAATAC, GAPDH F-ATGGGGAAGGTGAAGGTCG R-GGGGTCATTGATGGCAACAATA, PGR F-GACGTGGAGGGCGCATAT R-AGCAGTCCGCTGTCCTTTTCT. PCR was performed using SsoAdvanced Universal SYBR Green Supermix (BioRad, Hercules, CA, USA) with a CFX instrument (Biorad).

For RNAseq, RNA (3 replicates each of V, E2, or E2+MPA treated samples from donor 1 and donor 2) was DNAse treated and cleaned up using the RNeasy Mini kit (Qiagen, Hilden, Germany) or the RNA Clean and Concentrator 5 kit (Zymo, Irvine, CA, USA). RNA was submitted to the NIEHS sequencing core for library preparation using Illumina’s Ribo-Zero Gold kit for donor 1 RNA and Illumina Stranded mRNA Prep for donor 2 and paired-end sequencing using the Illumina Novaseq (75 nt reads, 60 million reads). Raw data were filtered to remove low-quality reads, mapped to hg38 using Tophat [29] and de-duplicated using Picard tools (2.18.15; “Picard Toolkit.” 2019. Broad Institute, GitHub Repository. https://broadinstitute.github.io/picard/ (accessed on 22 October 2018); Broad Institute, Cambridge, MA, USA). BAM files were imported into Partek Genomics Suite for analysis. RPKM for RefSeq genes was determined, and the maximum RPKM value from any sample for each gene (MAX) was listed. We noted differences between donor 1 and donor 2 datasets that reflect differences in RNA and library quality. For this reason, we analyzed the datasets from each donor separately and then compared the DEG so that each would be relative to its own V control. Genes for which the MAX < 1% of the mean RPKM for the whole dataset were filtered out (<0.02 for donor 1, <0.002 for donor 2). Values of 0 for V samples were replaced with a value of 0.05% of MAX to allow calculation of E2/V or (E2+MPA)/V. After PCA, it was noted that donor 1 V-1 and donor 1 E2-1 samples were outliers, potentially due to low reads, and were excluded from further analysis. Partek ANOVA was used to determine E2 vs. V, E2+MPA vs. V, and E2+MPA vs. E2 fold changes, and the List Manager function was used to find DEG (2-fold, FDR *p*-value < 0.05). Partek Venn diagrams were used to construct a list of all DEG in either donor to combine fold changes of the datasets for visualization. Data are deposited in GEO (GSE200807).

For HiC, frozen E2+MPA treated organoid samples were shipped to Active Motif (Carlsbad, CA, USA) for HiC and library preparation using the Arima HiC kit (Arima, San Diego, CA, USA). Libraries were sequenced by the NIEHS Genomics Core on the Illumina NovaSeq (50 nt reads, 800 million reads). The raw reads were mapped to human reference genome (GRCh38/hg38) using HiCUP version 0.7.1 [30]. Juicer version 1.8.9 was used to identify chromatin loops [31]. The A/B compartments were predicted using “eigenvector” function of Juicer at 100 Kb resolution. In order to find genes associated with ChIP peaks via looping, the mergePeaks function of HOMER [32] was used to find ESR1 peak overlaps with loop regions. Genes within loop ends were defined as their transcription start site (TSS) falling within either loop end and implemented in a Perl script. Genes within loop spans were found using the co-localize function of Easeq [33] to co-localize the hg38 geneset to the loop spans region set.

For PGR ChIPseq, crosslinked E2+MPA treated organoid cells were sent to Active Motif for Factor Path analysis using anti-PGR antibody sc-7208 (Santa Cruz Biotech, Santa Cruz, CA, USA). Resulting libraries were sequenced by the NIEHS Genomics Core On the Illumina NextSeq-High Output (50 nt reads, 40 million reads). Raw ChIP-seq reads were processed and aligned to the human reference genome hg38 using Bowtie [34]. The reads were de-duplicated, and peaks were called using MACS2 [35]. The mergePeaks function of HOMER was used to find shared PGR peaks in donor 1 and donor 2 organoid samples or for Venn analysis. Peak Annotation and Visualization (PAVIS; [36]) was used to compare the locations of PGR peaks relative to genes. Known motifs in PGR peaks were identified using HOMER findMotifs. Heatmap plots of PGR or ESR1 signals were created using EaSeq [33]. EaSeq was also used to locate the nearest gene TSS and TES to each PGR peak as a way to identify peaks within 100 kb of genes. Our previously published HiC from mouse uterus was analyzed to assign 100 kb bins to A or B compartments using Juicer. A or B compartment locations of ESR1 or PGR ChIP peaks were determined using GenomicRanges [37]. In order to find genes associated with ChIP peaks via looping, the mergePeaks function of HOMER was used to find ESR1 or PGR peak overlaps with loop regions. All new data are available on GEO (GSE200807). The following previously published sets were analyzed as part of the study: GSE132713 (endometrial PGR ChIPseq [18]), GSE69539 (hESC PGR ChIPseq [38]), mouse uterus GSE147843 (HiC [21]), GSE36455 (ESR1 and SMC1A ChIPseq [28,39]), GSE34927 (PGR ChIPseq [38])).

### 2.3. Western Blot

E2+MPA treated organoid cell pellets were homogenized in 50 mM Tris, pH 7.5, 150 mM NaCl, 1% Triton X-100, 2.5 mg Na Deoxycholate with added protease (cOmplete, Sigma Alrich, St. Louis, MO, USA) and phosphatase (Phosphatase Inhibitor Cocktails 2 and 3, Sigma) inhibitors. Protein levels were assayed using the BCA kit (Thermo Fisher, Waltham, MA, USA), and 12 µg per lane was loaded onto a Protean mini gel (10 well, 10%, Bio Rad Hercules, CA, USA) run, and transferred to nitrocellulose membrane using the Turbo-Blot transfer system (BioRad) according to manufacturer’s directions. Membrane was blocked with 5% milk (Santa Cruz Biotech, Santa Cruz, CA, USA) in TBST (20 mM Tris, pH 7.4 (Lonza, Morrisville, NC, USA), 140 mM NaCl (Lonza), 1% TWEEN-20 (Sigma). PGR was detected using a mixture of IMA5 12,658 (Invitrogen, Waltham, MA, USA), hPRa7 (kindly provided by Dr. Dean Edwards), and mAb 1294 (Santa Cruz; sc 166169) each at 1:1000 in milk overnight at 4C. Bands were detected using IRDye 800 CW Goat anti-Mouse IgG Secondary Antibody (Licor, Lincoln, NE, USA) diluted 1:20,000 in 5% milk for 45 min, and images generated using a Licor Fc instrument with Image Studio software (Licor). β-ACTIN was similarly detected using Actin (I-19)-R (Santa Cruz; sc-1616-R) diluted 1:5000, and IRDye^®^ 680RD Goat anti-Rabbit IgG Secondary Antibody diluted 1:20,000.

## 3. Results

### 3.1. Impact of Progesterone on Endometrial Organoid Genes

**Figure 1:**
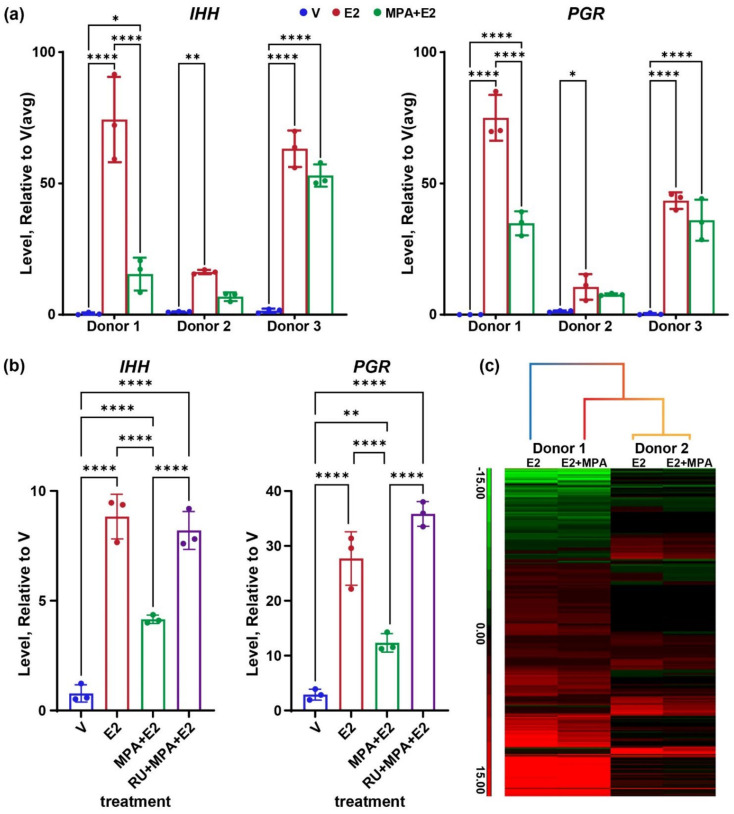
Progesterone attenuates estrogen-responsive genes. (**a**) RT-PCR for *IHH* or *PGR* from RNA after treatment of organoid isolated from 3 different donors with vehicle (V) estrogen (E2) or estrogen with medroxyprogesterone acetate (MPA+E2). *, **, **** *p* < 0.05, 0.01, and 0.0001, respectively, after one way ANOVA with uncorrected Fisher’s LSD test, *n* = 3 samples per group. (**b**) RT-PCR for *IHH* or *PGR* from RNA after treatment of organoid isolated from donor 2 with V, E2, MPA+E2, or RU486+MPA+E2. **, **** *p* < 0.01 and 0.0001, respectively after one way ANOVA with uncorrected Fisher’s LSD test, *n* = 3 samples per group. (**c**) Hierarchical cluster heatmap of E2 vs. V (E2) or E2+MPA vs. V (E2+MPA) differentially expressed genes (2-fold, FDR *p*-value < 0.05) of RNA from 2 different donors.

We previously described our use of endometrial organoids as an estrogen-responsive model to characterize the ESR1 transcriptome and cistrome in human endometrial epithelial cells [26]. To test the progesterone response of the organoids, we added analysis of organoids that were treated with estradiol (E2) to increase expression of PGR and then treated with E2 together with the progesterone agonist, medroxyprogesterone acetate (MPA). PGR is detected by Western blot of protein isolated from E2+MPA treated organoids (Figure S1a), confirming the expression of the protein in the organoids. The MPA treatment resulted in the inhibition of estrogen induction of the Indian hedgehog (*IHH*) transcript in organoids derived from two of three different donors (Figure 1a). *PGR* transcript levels manifested a similar pattern of estrogen induction and MPA inhibition (Figure 1a). Additionally, co-treatment with the PGR antagonist RU486 results in reversal of the MPA inhibition of both *IHH* and *PGR* (Figure 1b), indicating the MPA suppression is PGR dependent. For subsequent comprehensive analysis of transcriptomes and cistromes, we focused on samples from donors one and 2 to include a variation between individuals. RNAseq was then used to assess the global impact of MPA on the organoid transcriptome. We compared differentially expressed genes (DEG; 2-fold, FDR *p* < 0.05) of E2 vs. V or E2+MPA vs. V of donor one or two derived organoids (Figure S1b and Figure 1c).

We previously observed that donor two derived organoids were less responsive to estrogen than donor one derived organoids [26]. Similarly, E2+MPA produces fewer DEG from donor two (Figure S1b, Table S1a,b). Hierarchical clustering shows that donor two MPA+E2 vs. V DEG clusters together with donor two E2 vs. V DEG. For donor one, a pattern of attenuation of fold change caused by the inclusion of MPA is evident (Figure 1c). Differential expression analysis between E2+MPA and E2 samples (2-fold, FDR *p* < 0.05) yielded 102 genes (Figure S1b, Table S1c,d), with most being examples of attenuation of estrogen response (Table S1e), suggesting that beyond inhibiting estrogen, the organoids are minimally responsive to progesterone.

### 3.2. Progesterone Receptor Cistrome of Organoids

**Figure 2:**
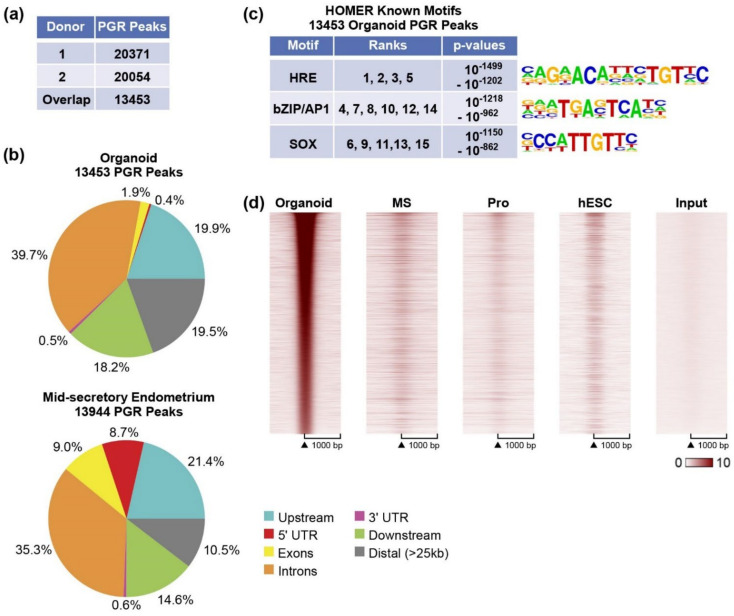
PGR cistrome of organoids. (**a**) Summary of PGR peaks in both donors; (**b**) PAVIS analysis showing locations of PGR peaks in mid-secretory endometrium and organoids. (**c**) HOMER known motif analysis. Summaries of the top 15 HOMER known motifs enriched in PGR peaks. The ranks of each motif, as determined by *p*-value, along with the range of *p*-values covered by the ranks are indicated. Motif logos are shown. (**d**) Heatmap indicating PGR ChIP signal centered on PGR locations (±1 kb) in organoids. MS: mid-secretory endometrium, Pro: proliferative endometrium, hESC: human endometrial stromal cells, Input: organoid input.

PGR was present (Figure 1a and Figure S1a), yet MPA primarily attenuated estrogen responses and did not appear to independently regulate many genes (Figure 1c, Table S1). To assess PGR interaction with organoid chromatin, we examined the PGR cistrome of organoids treated with MPA+E2 and compared it to the PGR cistrome of mid-secretory endometrium [18]. Similar numbers of PGR peaks were identified when using organoids from either donor (Figure 2a). For downstream analysis, we focused on the 13,543 PGR peaks common to both donors. When we looked at the locations of the PGR peaks relative to annotated RefSeq genes, 42% of the PGR peaks were located at gene bodies (introns, exons, 5′ UTR, and 3′ UTR; Figure 2b).

This is contrary to previously published observations in mid-secretory and proliferative endometrial tissue, where the majority of PGR occupying sites were found in the gene body [18]. To ensure a comparable analysis, we re-processed the endometrial biopsy PGR ChIPseq data using the same pipeline used for the organoids and found that 53.6% of the mid-secretory PGR peaks were at genes (Figure 2b and Figure S2a), and fewer peaks were located distally (>25 kb 19.5% of organoid and 10.5% of endometrium; Figure 2b). A similar distribution occurs in proliferative samples [18], indicating the PGR in organoids is interacting in more distal regions than the PGR does in the whole endometrium. We analyzed sequences at organoid PGR binding sites for enriched motifs, and since our previous study focused on differentially enriched PGR peaks of the endometrial biopsies [18], we also included a re-analysis of motifs in mid-secretory endometrium. HRE motifs were the most highly enriched in the mid-secretory endometrium PGR peaks (Figure S2b) and organoid samples (Figure 2c), followed by bZIP/AP1 motifs. Unlike the endometrial PGR peaks, we also observe SOX motif enrichment in the organoid PGR peaks (Figure 2c).

**Figure 3:**
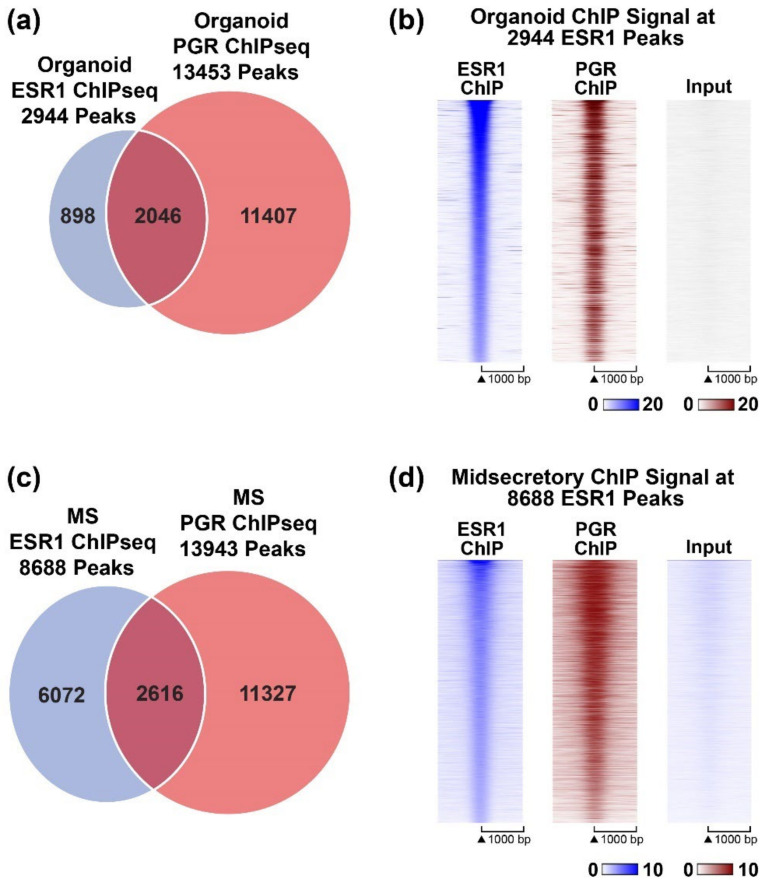
PGR and ESR1 ChIP peaks in organoids and in mid-secretory endometrium. (**a**) Comparison of locations of ESR1 and PGR peaks from organoids; (**b**) Heatmap indicating organoid ESR1 or PGR ChIP signal centered on ESR1 peak locations (±1 kb) in organoids. (**c**) Comparison of locations of ESR1 and PGR peaks from mid-secretory endometrium (**d**) Heatmap indicating mid-secretory endometrium ESR1 or PGR ChIP signal centered on ESR1 peak locations (±1 kb) in mid-secretory endometrium.

We have thus far observed progesterone attenuation of estrogen response in organoids and altered localization of PGR peaks relative to genes in organoids vs. endometrial biopsies. To further investigate the PGR occupancy pattern of the organoids compared to endometrium samples, we compared the ChIPseq signal of organoid PGR peaks to that from mid-secretory and proliferative endometrium at the same locations. We also included PGR peaks from a previously reported study using estrogen, progesterone, and cAMP-treated human endometrial stromal cells (hESC [38]). This analysis reveals that the organoid PGR peaks are not at locations observed in the endometrium or hESCs (Figure 2d), which likely impacts its ability to regulate endometrial genes. Since we also observed that the addition of MPA mainly attenuated estrogen responses, we compared PGR and ESR1 peaks within organoids or endometrial biopsies. In organoids, the PGR peaks overlap 70% of the ESR1 peaks (Figure 3a,b). By examining the signal intensity centered on ESR1 peak locations, we observe that PGR interaction is co-localizing at ESR1 binding locations (Figure 3b). In mid-secretory endometrium, PGR peaks overlap a smaller proportion of ESR1 peaks than seen in organoids (mid-secretory PGR peaks overlap 30% of ESR1 peaks Figure 3c,d), another indication that the epithelial organoid PGR cistrome differs from that of the endometrium.

### 3.3. Chromatin Structure of Epithelial Cells

#### 3.3.1. Enhancer-Gene Interactions in Organoids

**Figure 4:**
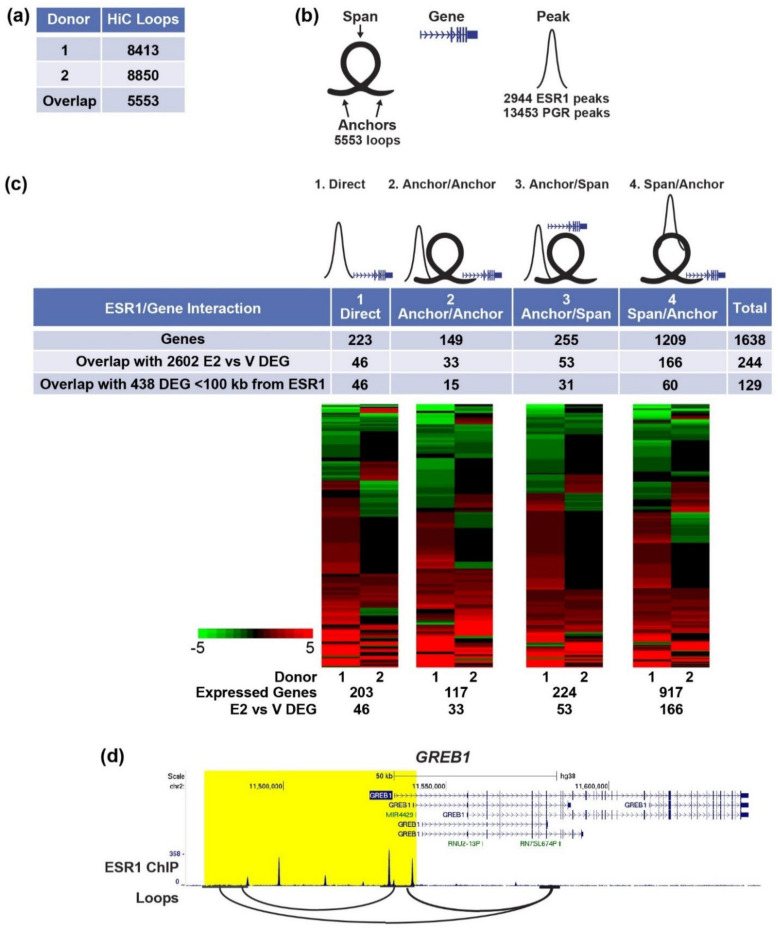
Analysis of ESR1-Gene Interactions Using HiC Loops. (**a**) HiC loops in each donor and overlapping loops; (**b**) Illustration of terms used in HiC ESR1/PGR ChIPseq and gene integration analysis to represent loops, with anchors with a connecting span, genes and ChIPseq peaks. (**c**) Genes in the four types of ESR1 peak-gene interactions described in the text. Heatmaps of fold E2 vs. V of all genes in each group expressed in either donor, with numbers of genes expressed and differentially expressed indicated below each. (**d**) In organoids, *GREB1* is an example of direct, anchor/anchor and span/anchor ESR1-gene interaction.

The 3-dimensional structure of chromatin facilitates interactions between distal enhancer regions that bind transcription factors, including ESR1 or PGR, and the genes they regulate. Using HiC, we analyzed loops in organoid chromatin and found a comparable number of loops from either donor (Figure 4a). We utilized the 5553 loops shared by the two donors to find PGR or ESR1 binding regions that interact with genes. For this analysis, we divided the loops into left and right “anchors” (Figure 4b; regions at the left or right ends of each loop) and “spans” (the region between the end of the left anchor and the start of the right anchor; Figure 4b). We described four different ways in which an ESR1 or PGR peak could interact with a gene.

First, ESR1/PGR can interact directly with a gene promoter (Figure 4c, “direct”). Second, ESR1/PGR can interact with the anchor region of a loop and be brought into proximity by the loop to a gene on the opposite anchor (Figure 4c, “anchor/anchor”). Third, ESR1/PGR can interact with the anchor region of a loop in which there are genes in the span of that loop (Figure 4c, “anchor/span”). Finally, ESR1/PGR can interact with the span region of the loop in which there are genes in the anchor region(s) (Figure 4c, “span/anchor”). The annotated genes associated with each of the four types of ESR1/gene or PGR/gene arrangements were compiled, and hormone-regulated organoid genes were assigned to interacting sites (Tables S2 and S3). Very few (15) of the 102 genes regulated by MPA+E vs. E2 (Figure S1b) were assigned to enhancers (Figure S3, Table S3). Because the organoids were predominantly estrogen-responsive and so few of the PGR/gene interactions identified this way were regulated, we focused on the estrogen-responsive ESR1/gene pairs. Analysis of estrogen-regulated organoid genes reveals that 46 of the 223 direct genes (Figure 4c, Table S2a), 33 of 149 anchor/anchor genes (Figure 4c and Table S2b), 53 of 255 anchor/span genes (Figure 4c and Table S2c), and 166 of 1209 span/anchor genes (Figure 4c and Table S2d) are regulated by estrogen in the organoids. Heatmaps (fold E2/V) of the genes identified for each type of ESR1/gene interaction that are expressed in organoids from either donor indicates both up- and down-regulated transcripts in all types. The number of E2 vs. V DEG in the four groups together is 244 genes, as some genes have more than one type of ESR1/gene interaction. For example, the *GREB1* gene is in the direct, anchor/anchor, and span anchor/groups (Figure 4d), as there are ESR1 peaks at the TSS, another ESR1 peak on a distal loop anchor that interacts with the TSS, and more ESR1 peaks within a loop span as well (Figure 4d).

We compared the approaches of 1: combining ChIPseq and chromatin structure to find candidate E2/ESR1 regulated genes and 2: assigning candidate genes based on the gene closest to ESR1 peaks within 100 kb (438 genes, [26]) by determining how many of the DEG were captured by either method. For the directly interacting genes, which do not consider 3D structure, either method is the same, and all 46 genes were identified (Figure 4c). In the other three groups, the closest gene analysis results in fewer genes (15 of 33 anchor/anchor genes, 31 of 53 anchor/span genes, 60 of 166 span anchor genes Figure 4c) overall identifying 119 of 244 genes, or about 50%, of the four combined interaction groups. This reveals that considering chromatin interactions can significantly improve the identification of genes regulated by distal enhancers interacting with ESR1.

#### 3.3.2. Compartment Localization of PGR and ESR1 Peaks

**Figure 5:**
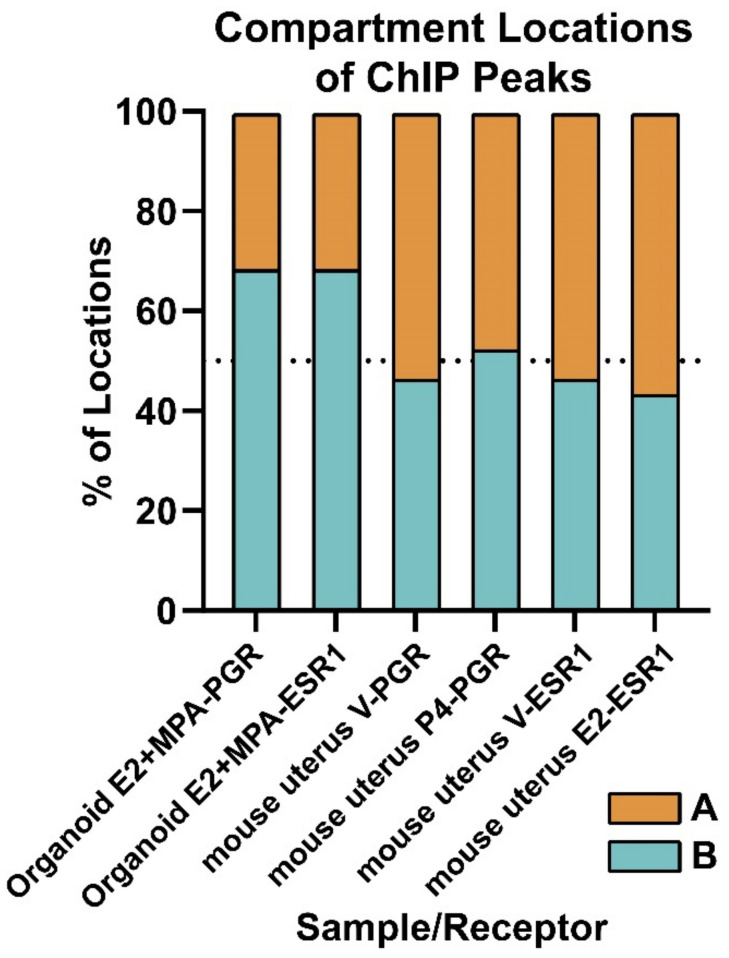
A higher proportion of organoid PGR and ESR1 ChIPseq peaks co-localize to B (inactive) compartment than is seen in mouse uterus.

Because we see evidence that PGR is located more distally from genes, is not co-localizing with whole endometrial PGR sites, and the organoids are insensitive to progesterone response, we investigated the locations of the PGR peaks relative to the A (active chromatin) and B (inactive chromatin) compartments. This analysis reveals that most PGR peaks (68%) are co-localized in the B compartment (Figure 5). For comparison, we analyzed organoid ESR1 peaks and found that 68% of ESR1 peaks are within the B compartment as well (Figure 5). Next, we analyzed mouse uterus PGR peaks and ESR1 peaks relative to A and B compartments from previously published ChIPseq and HiC datasets [17,21,28,39] and found that in mouse uterine tissue, fewer ESR1 and PGR peaks (43–52%) co-localize with B the compartment (Figure 5). This analysis suggests that in our organoid model, PGR and ESR1 are not only more distal from genes than in the whole endometrium but also more localized to B (inactive) compartment regions of chromatin, which may attenuate gene responses to hormones.

The proportion of organoid of mouse uterus PGR or ESR1 peaks that co-localize with A or B compartments identified in HiC from the same sample and treatments are shown.

### 3.4. Chromatin Landscape of IHH and PGR Likely Mediates Their Hormone Regulation

ESR1 and PGR peaks and loops in the vicinity of two genes, *IHH* and *PGR*, are shown as examples. Both genes were increased by estrogen and showed attenuated inductions from estrogen+MPA (Figure 1a). ESR1 and PGR peaks [18] and HiC interactions relative to regions that potentially mediate the hormone regulation of *IHH* and *PGR* genes in organoid and endometrium are shown. Additionally, PGR peaks in human endometrial stromal cells (hESC) treated with estrogen, progesterone, and cAMP were included [38].

Some of the observations regarding organoid vs. endometrial ESR1 and PGR cistromes are illustrated in the specific example of *PGR*. In organoids, the *PGR* gene is within a 450 kb loop (Figure S4a). Multiple ESR1 and PGR peaks are apparent within this looped region. There are fewer mid-secretory endometrium ESR1 peaks than proliferative endometrium ESR1peaks that are smaller and are at locations coinciding with ESR1 proliferative peaks (Figure S4a). The organoid ESR1 peaks occur at some but not all positions that coincide with ESR1 peaks in proliferative endometrium (Figure S4a), illustrating the observation we previously reported that the organoid ESR1 cistrome most closely resembles that of proliferative endometrium [26]. PGR peaks of mid-secretory and proliferative endometrium mostly coincide, with more PGR peaks in mid-secretory endometrium. There are few hESC PGR peaks, and they mostly co-occur with proliferative and mid-secretory endometrium PGR peaks. In this *PGR* gene loop, there are more PGR peaks in organoids than proliferative or mid-secretory endometrium. Not all organoid PGR peaks co-localize with endometrial PGR peaks. There are more PGR peaks than ESR1 peaks in organoids; all the organoid ESR1 peaks are at the locations with an organoid PGR peak. Multiple organoid PGR peaks do not coincide with PGR or ESR1 peaks in the endometrium. Overall, the chromatin near the *PGR* gene illustrates the observation that the organoid PGR cistrome does not resemble those of endometrium or hESCs (Figure 2d).

PGR and ESR1 ChIPseq [21,38,39] from uterus of ovariectomized mice treated with vehicle, progesterone, or estrogen in the region near the *Pgr* gene (Figure S4b) was compared to that of human endometrium (Figure S4a). We included a summary of HiC loops [21,28], as well as ChIPseq of the cohesin subunit SMC1A [21,28], which often coincides with loop ends. The uterine chromatin landscape near the mouse Pgr gene is arranged similarly to that of humans, although the gene direction is opposite (human *PGR* is 3′ to 5′′, mouse *Pgr* is 5′ to 3′). Since the mouse HiC analysis involves whole uterine tissue, a more complex looping pattern occurs, with multiple interactions in the region, but similar to the human gene, the loops in the region incorporate >400 kb. There is intense PGR and ESR1 binding to multiple sites, especially at the 3′ end of the *Pgr* transcript (Figure S4b), similar to the PGR and especially ESR1 peaks at the 3′ end of human *PGR* (Figure S4a). The ESR1 peaks in both humans and mice increase in size and number in proliferative (estrogen dominant) endometrium (Figure S4a) and estrogen-treated uterus (Figure S4b), consistent with estrogen-dependent ESR1 recruitment.

In addition to the *PGR* gene, we also observed that the chromatin landscapes of the human and mouse endometrial *IHH* genes are quite similar. In organoids, the *IHH* gene interacts with distal regions via two loops (Figure S4c), similar to loops connecting a region 63 kb 5′ of the mouse *Ihh* gene to the *Ihh* TSS or to a region 12 kb 5′ of *Ihh* (Figure S4d). The loop anchors in the mouse also bind SMC1A. Two ESR1 and PGR peaks are apparent at 20 kb 5′ of the *IHH* TSS in organoids, as well as in the proliferative phase endometrium (Figure S4c). Previous work described a similar region at 19 kb 5′ of the mouse uterus *Ihh* gene that binds PGR and ESR1, with demonstrated hormone-dependent enhancer activity (Figure S4d and [19]). A PGR peak occurs at 70 kb 5′ of the *IHH* TSS in organoids, in mid secretory endometrium, and hESC (Figure S4c), while an ESR1 peak is found here in proliferative endometrium but not in mid secretory endometrium or organoids. Although more proximal, in the mouse, PGR similarly binds 39 5′ of *Ihh* (Figure S4d). PGR and ESR1 bind 100 kb 5′ of the *IHH* gene in mid secretory as well as proliferative endometrium, but not in organoids or hESC (Figure S4c). In the mouse uterus, PGR binds 63 kb 5′ of *Ihh*, and ESR1 binds 25 kb and 10 kb 5′ of *Ihh* (Figure S4d). The similar loops and ESR1 and PGR cistromes near PGR and IHH mouse and human genes align with their estrogen and progesterone responses in both species.

## 4. Discussion

### 4.1. Progesterone Attenuates Estrogen Gene Response of Endometrial Epithelial Organoids

We utilized the endometrial epithelial cell organoid model as a way to study mechanistic details of hormone response intrinsic to epithelial cells. We observed a robust transcriptional response to estrogen treatment and were able to characterize the ESR1 cistrome and compare it to the whole endometrium in a previous study [26]. Here, we examined the progesterone responsiveness of the organoid model. At the transcript level, PGR expression was quite low without estrogen treatment (Figure 1a) but was robustly increased by estrogen and readily detected by Western blot (Figure 1a and Figure S1a). Despite this, the primary transcriptional impact of progesterone treatment was attenuation of the estrogen response (Figure 1a,c). Progesterone has an important biological role in controlling the extent of estrogen response, especially within epithelial cells, where PGR-mediated growth arrest is characteristic of receptivity [40]. PGR expression decreases, and FOXO1 trans-locates into the nucleus, where it inhibits cell cycle progression [8]. This mechanism is evident in the observed hyper-responsiveness of uterine tissue from PGR-null mice [9] and persistent epithelial proliferation when PGR is deleted from epithelial cells [10,11], preventing embryo attachment and implantation. PGR and ESR1 sites that overlap could potentially mediate progesterone attenuation of estrogen responses by competing for binding of PGR or ESR1. Differences we observed between PGR cistromes of organoids, hESC and whole endometrium will also include epithelial or stromal enrichment of organoids and hESC, respectively, but may also be indicative of the progesterone insensitivity of the organoids.

In a recent study, activation of NOTCH and inhibition of WNT signaling in organoids was a requirement for the cells to acquire a secretory phenotype after progesterone treatment [41]; other work has shown estrogen treatment together with NOTCH inhibition enhances ciliagenesis in organoids [42]. Our culture conditions include WNT activators and estrogen, which may restrict the impact of progesterone response by preventing the transition from ciligenesis to a secretory state and could explain why PGR peak locations do not correspond to those in the endometrium (Figure 2d). Accordingly, assessing the impacts of NOTCH and WNT signals in culture conditions on the PGR cistrome- and progesterone-induced responses will be an important focus of future work. The DNA motifs enriched in organoid PGR peaks matched those of mid-secretory endometrium PGR peaks (HRE, bZIP1/AP; Figure 2c and Figure S2b), indicating the DNA binding domain specificity of the organoid PGR is appropriate and does not account for the restriction of progesterone response to attenuation of estrogen response. Organoid PGR ChIPseq peaks were additionally enriched for SOX motifs, which likely reflects the selective epithelial expression of SOX17 [19], allowing for enrichment to be observed.

### 4.2. Localization of PGR and ESR1 in the B (Inactive) Compartment of Organoid Chromatin

The higher proportion of B (inactive) compartment localization of PGR in organoids correlated with progesterone’s limited activity. However, ESR1 is similarly localized in the B compartment but mediates gene responses [26]. PGR is more evenly distributed between A and B compartments in ovariectomized mouse uterus (Figure 5), where PGR expression is restricted to epithelial cells. A recent study showed that PGR and ESR1 peaks in Ishikawa endometrial cancer cells are mostly in the A (active) compartment (79% and 83%, respectively) [22]. These observations in mouse uterus and in Ishikawa cells indicate that B compartment sequestration of PGR is not a general characteristic of epithelial cells. Organoid PGR co-localizes with 70% of ESR1 peaks (Figure 3a), whereas in mid-secretory endometrium, PGR peaks co-occur with a smaller proportion of ESR1 peaks (30%, Figure 3c), suggesting ESR1/PGR co-localization in organoids may reflect an inability of PGR to access the regions of chromatin observed in endometrial samples. Rather, PGR interacts with sites made accessible by ESR1 binding. In our previous study, we observed that organoid ESR1 peak locations were similar to those of proliferative endometrium [26], whereas here, we observed that PGR peak locations differed from endometrial samples (Figure 2d). This may be the key difference allowing estrogen response, but not progesterone response, despite both ESR1 and PGR localization in the B compartment and more distal from genes. Therefore, the current culture condition of endometrial organoids is not sufficient to investigate PGR regulation of gene expression. Either combining organoids with stroma cells or improving the culture conditions may be required.

### 4.3. Chromatin Structure Reveals Enhancer-Gene Interactions

PGR and ESR1 often bind DNA sites in enhancers that are not close to the genes they regulate but interact with genes via loops in the 3D structure of the chromatin. One approach often used to “assign” enhancer-gene interaction is to find the closest gene within a maximum kb cutoff. We used HiC to identify chromatin loops and then describe interactions of ESR1 or PGR binding peaks relative to loops (Figure 4 and Figure S3). We located genes within these interacting sites and used our organoid RNAseq datasets to find those that are expressed and whether genes interacting with ESR1 or PGR were regulated by E2 (E2 vs. V DEG) or MPA (MPA+E2 vs. E2), respectively. We identified both PGR and ESR1 enhancer-gene interactions, but because progesterone response was minimal in the organoids, this approach yielded just 15 regulated genes. By incorporating the organoid HiC analysis to locate potential interactions between ESR1 peaks and estrogen-regulated genes, we were able to find twice as many ESR1-gene interactions than were inferred using DEG <100 kb from ESR1 peaks (Figure 4c), which greatly increases the significance of our analysis. Overall, considering chromatin structure is advantageous in defining enhancer-gene interaction, but in this study, analysis for PGR targets is impacted by the limited progesterone response. We observe similarities between mouse and human cistromes and loops near hormone-responsive genes, such as *PGR* and *IHH* (Figure S4), suggesting that enhancers involved in their regulation are conserved. Potential enhancers and chromatin structures identified in this study can be further evaluated in mice and in human in vitro systems (organoids and hESCs).

## 5. Conclusions

In sum, our study describes transcriptional profiles, the PGR cistrome, and the three-dimensional chromatin structure of hormone-treated endometrial epithelial cells cultured as organoids. Although progesterone primarily led to attenuation of estrogen-regulated genes, future work will investigate whether altered culture conditions might increase progesterone responsiveness. The ability to control hormone responsiveness in this in vitro model will advance our understanding of mechanisms involved in processes intrinsic to endometrial epithelial cells.

## Data Availability

RNAseq, ChIPseq and HiC data are available from GEO accession GSE200807.

## Supplementary Materials

The following supporting information can be downloaded at: https://www.mdpi.com/article/10.3390/cells11111760/s1, Figure S1: Expression of PGR protein in organoids and summary of RNAseq differentially expressed genes; Figure S2:PGR ChIPseq analysis of human endometrium; Figure S3: Genes in the four types of PGR peak-gene interactions; Figure S4: PGR and ESR1 peaks and chromatin interactions near human and mouse IHH and PGR genes; Table S1: differentially expressed genes, MPA+E2 vs. V in donor 1 (a) and donor 2 (b); E2+MPA vs. E2 in donor 1 (c) and donor 2 (d) or donor 1+2 (e); Table S2: E2 vs. V of genes that are expressed in organoids that fall into groups 1–4 as described in Figure 4. Table S3 15 E2+MPA vs. E2 DEG in to groups 1–4 described in Figure S3.
