# Supplementary material for: Progesterone Signaling in Endometrial Epithelial Organoids
Source: Cells. 2022 May 27;11(11):1760. doi: 10.3390/cells11111760 (PMC9179553; doi:10.3390/cells11111760)
Supplement: Supplementary file 1 [file cells-11-01760-s001.zip › Supp Fig legends.pdf]

Supplemental Figure Legends

Figure S1 Expression of PGR protein in organoids and summary of RNAseq differentially expressed genes.

a. Western blot of proteins isolated from E2+MPA treated organoids showing PGR-B and PGR-A and β-ACTIN bands (arrowheads). Sizes of molecular weight markers, in kd, are indicated
b. Summary of E2 vs V, E2+MPA vs V and E2+MPA vs E2 differentially expressed genes from donors 1 and 2.

Figure S2 PGR Chisel analysis of human endometrium

a. Comparison of locations of proliferative and mid-secretory PGR peaks.
b. HOMER known motif analysis. Summaries of the top 15 HOMER known motifs enriched in PGR peaks from mid-scretory endometrium. The ranks of each motif, as determined by p-value, along with the range of p-values covered by the ranks are indicated. Motif logos are shown.

Figure S3: Genes in the four types of PGR peak-gene interactions are described in the text.

Figure S4. PGR and ESR1 peaks and chromatin interactions near human and mouse *IHH and PGR* genes. For the human genes, PGR and ESR1 ChIPseq from mid secretory (MS) and proliferative (Pro) whole endometrium and from organoids is shown along with hESC PGR ChIPseq. For the mouse genes PGR, ESR1, and SMC1A from V, P4 or E2 treated whole uterus is shown. Interacting loops from human organoid or mouse uterus HiC are also shown.

a. Human *PGR*. Gene is highlighted in blue.
b. Mouse *Pgr.* Gene is highlighted in blue.
c. Human *IHH*. The transcript is highlighted in blue, regions with ESR1 peaks at 20kb 5′ of the *IHH* transcription start site (TSS) are highlighted in grey, at 70kb 5′ of *IHH* are highlighted in purple and at 100 kb 5′ of *IHH* are highlighted in green.
d. Mouse *Ihh*. Regions comparable to the human gene are highlighted as described in c.
