# Supplementary figures and images for: Progesterone Signaling in Endometrial Epithelial Organoids
Source: Cells. 2022 May 27;11(11):1760. doi: 10.3390/cells11111760 (PMC9179553; doi:10.3390/cells11111760)

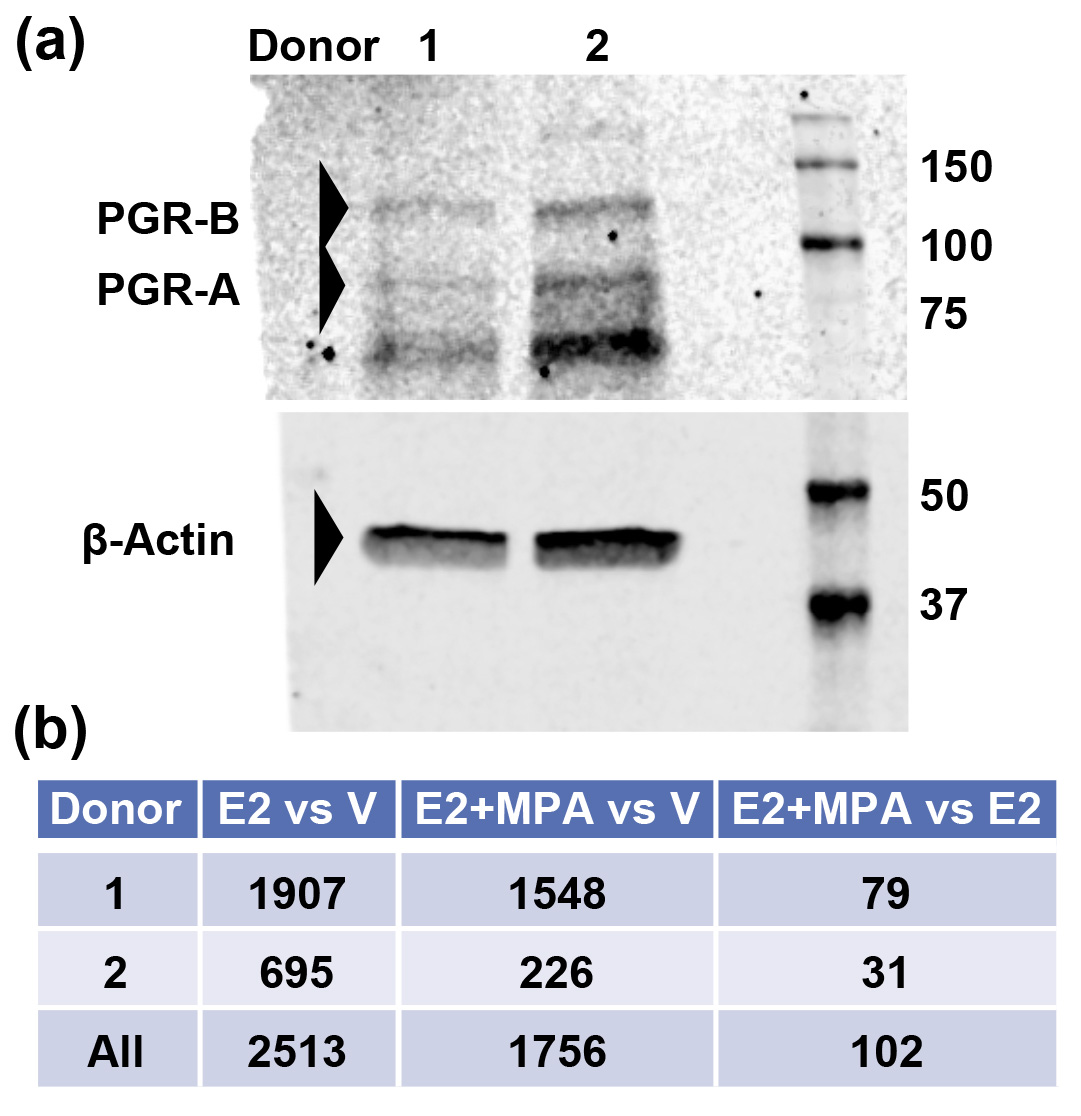

Supplement: Supplementary file 1 [file cells-11-01760-s001.zip › HewittS22_1842 Fig S1 v02 3w x 3.1h 360dpi.jpg]

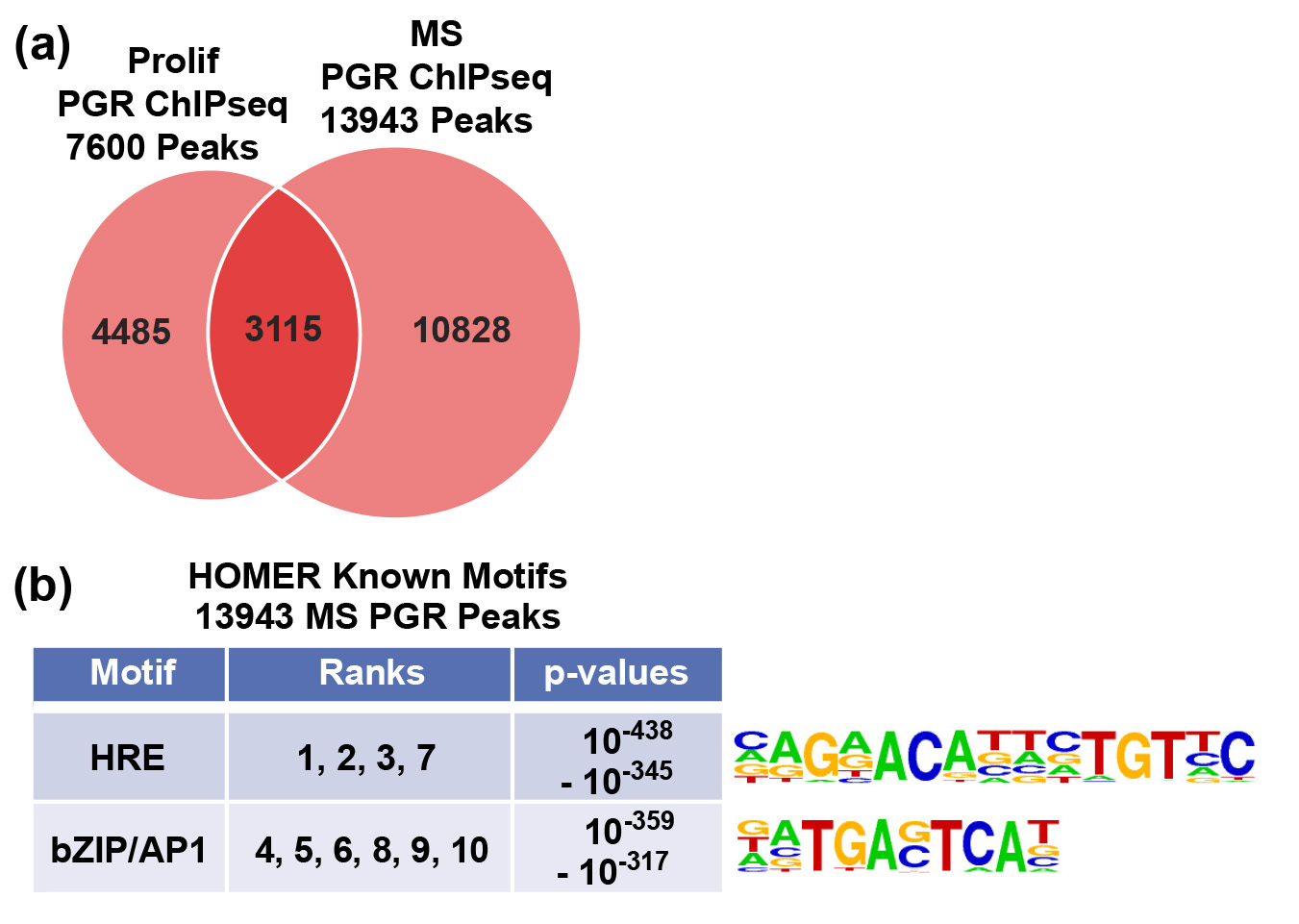

Supplement: Supplementary file 1 [file cells-11-01760-s001.zip › HewittS22_1842 Fig S2 v01 4.5w x 3.2h 300dpi.jpg]

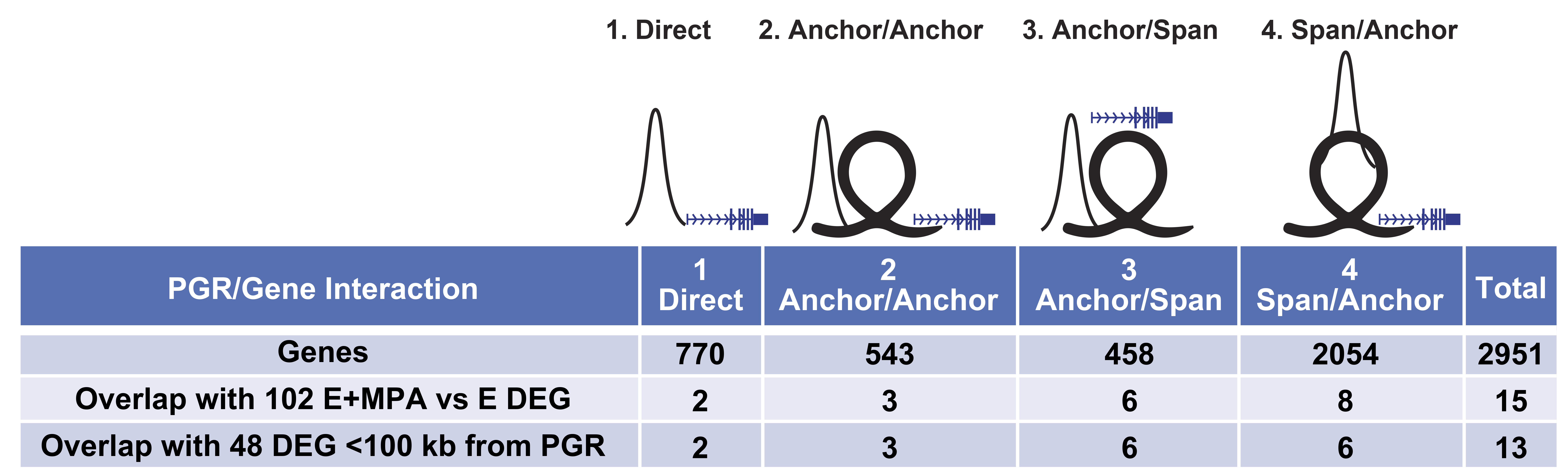

Supplement: Supplementary file 1 [file cells-11-01760-s001.zip › HewittS22_1842 Fig S3 v02 7.25w x 2.2h 1200dpi.jpg]

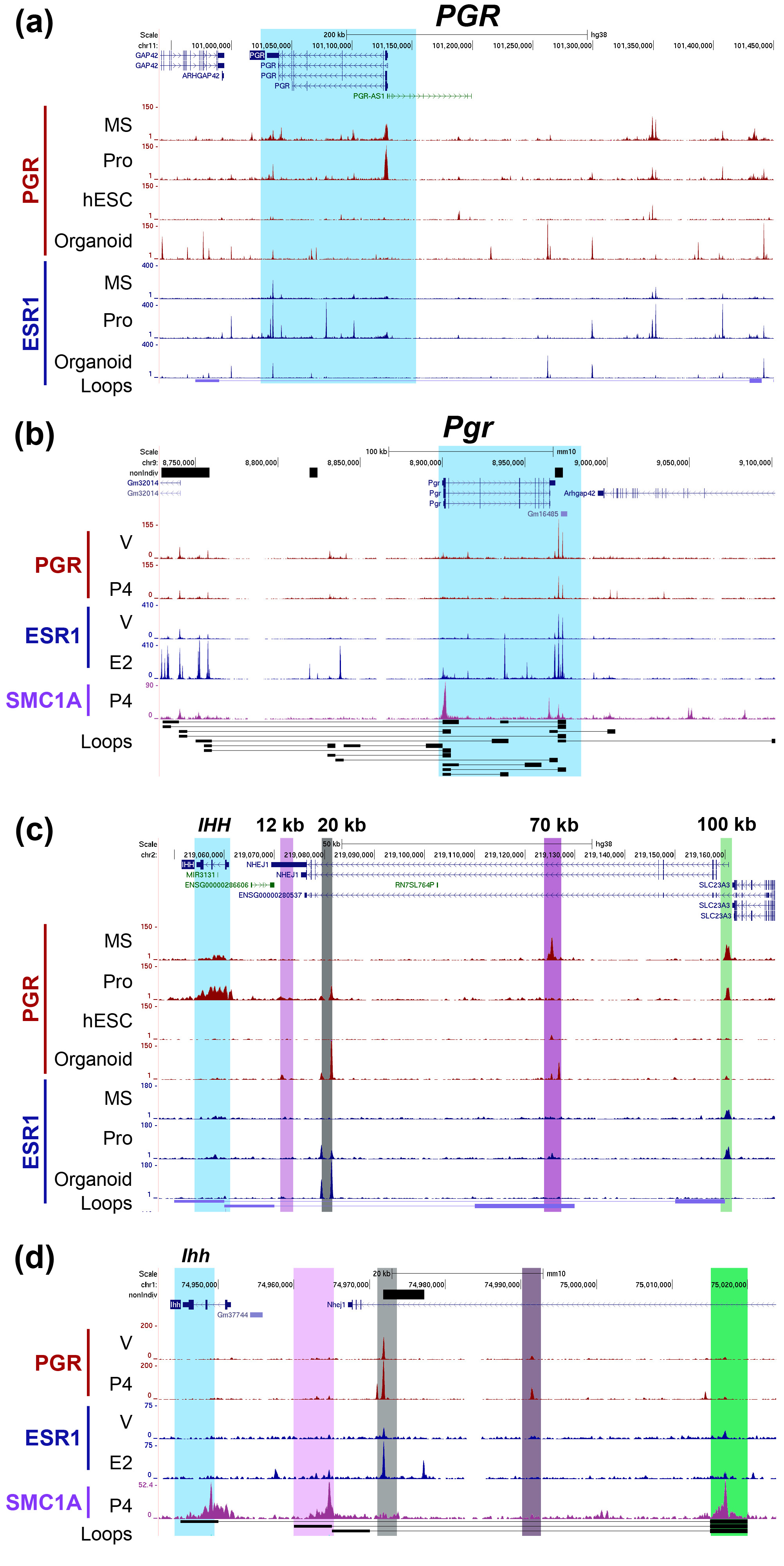

Supplement: Supplementary file 1 [file cells-11-01760-s001.zip › HewittS22_1842 Fig S4 v02 4w x 8h 500dpi.jpg]
